# A Case of Jejunal Atresia Associated With Heterotrophic Pancreas and Meckel’s Diverticulum

**DOI:** 10.7759/cureus.32766

**Published:** 2022-12-21

**Authors:** Chava Aravind Kumar, Chandrashekhar Mahakalkar

**Affiliations:** 1 General Surgery, Datta Meghe Institute of Medical Sciences, Wardha, IND

**Keywords:** pediatric bowel obstruction, meconium ileus, heterotrophic pancreas, meckel´s diverticulum, jejunal atresia

## Abstract

Apart from meconium ileus, amniotic fluid plug syndrome, malrotation of the gut, Hirschprung’s disorder, trauma, and other rare causes, bowel atresia is one of the most common causes of bowel obstruction in newborns. Jejunal atresia can affect multiple lengths of the bowel. The higher the level of atresia, the greater the severity. The outcome of bowel atresia related to surgical repair is favorable. In general, both mortality and morbidity are affected by affiliated medical conditions such as preterm birth, cystic fibrosis, and other congenital anomalies; the sophistication of the lesion; and surgical complications. We present the case of a one-day-old baby who had two episodes of bilious vomiting with abdominal distension within 10 minutes of birth. The baby was advised to undergo ultrasonography of the abdomen and pelvis for further evaluation, and the findings were reported.

## Introduction

Jejunal atresia can affect multiple lengths of the bowel. The severity increases with the level of atresia. The consequence of bowel atresia because of surgical repair is favorable [1]. Atresia can be divided into five traditional categories [2]. Type I atresia is defined by luminal webs or membrane proteins with mural continuity. Type II atresia is defined by two blind ends connected by a fibrous cord. Type IIIa atresia has a mesenteric gap and two unrelated ends. Type IIIb atresia is distinguished by two separable ends, a substantial mesenteric defect, no proximal small intestine mesentery, and anterior jejunal atresia. Numerous segments of hypoplasia are a feature of type IV atresia and type V stenosis [3,4].

When analyzed anatomically, an ectopic pancreas is a pancreatic tissue that is abnormally located and lacks morphological or vascular continuity with the normal tissue. It has an overall prevalence of 1% to 13% but rarely affects newborns. An endodermal pancreas is most typically found in the intestinal system (25%-38%), a portion of the small intestine (17%-36%), and the jejunum (15%-21%). It is also found in the liver, Meckel’s diverticulum, navel, spleen, and reproductive organs [5]. When no complications arise, it is mostly symptomless and is thus often only identified accidentally during surgical procedures or imaging examinations. An ectopic pancreas can result in pancreatitis, hepatocellular pseudocyst, tumor progression, gastrointestinal hemorrhage or atresia, and intussusception in some cases [6].

Meckel's diverticulum is one of the most common birth gastrointestinal anomalies, with an occurrence of 1% to 3%, but it affects 4% to 6% of infants aged below two years [7]. In 1809, Friedrich Meckels established its embryonic origin, which is caused by imperfect regression of the omphalomesenteric duct all through gestational weeks 5 to 7 [8]. It is undiagnosed in most cases, but health problems could also include inflammatory response, internal bleeding, and bowel obstructions. Meckel's diverticulum is usually seen in children who do have hemorrhage and intussusception when the symptoms first appear. In adults, Meckel's diverticulum can cause intestinal obstruction and inflammation [9]. Although the annular pancreas and Meckel's diverticulum are rare genetic intestinal abnormalities, their occurrences in infants are uncommon [10].

## Case presentation

**Figure 1:**
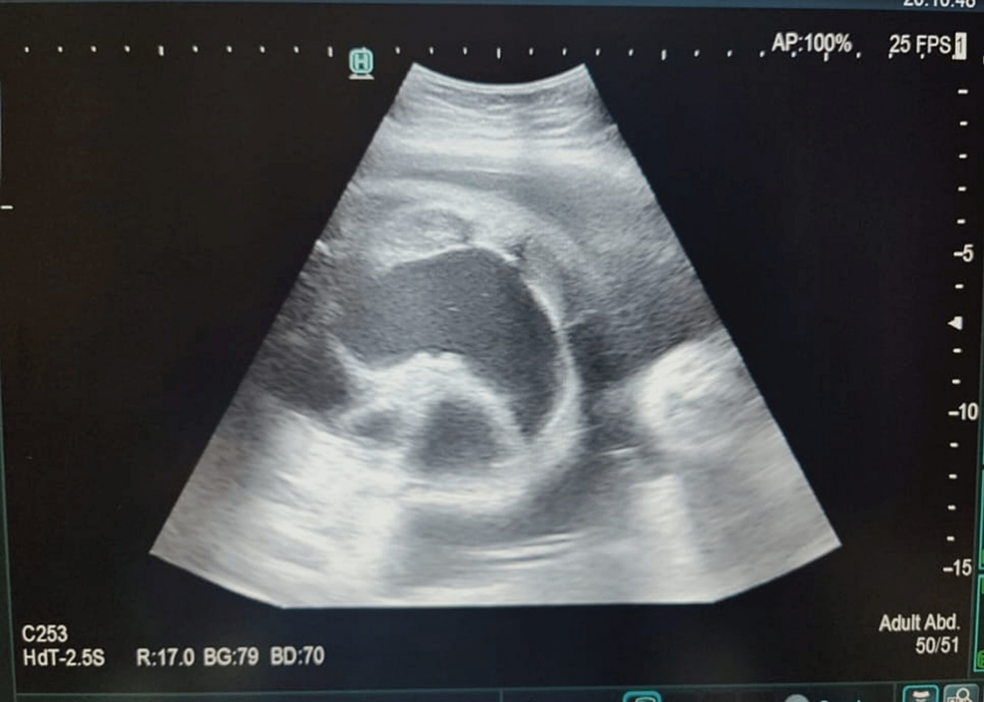
Antenatal scan showing dilated bowel loops.

A one-day-old male baby was delivered by full-term cesarean section, with a significant antenatal history, and was suggestive of dilated bowel loops in the antenatal scan (Figure 1). History of consanguineous marriage is present. The newborn cried immediately after birth. The first feed was given after two hours after birth, and within 10 minutes, the baby had two episodes of bilious vomiting with abdominal distension and no meconium passed. On further evaluation using erect abdominal radiography, multiple proximal gas shadows were observed, with no gas shadows beyond the obstruction.

**Figure 2:**
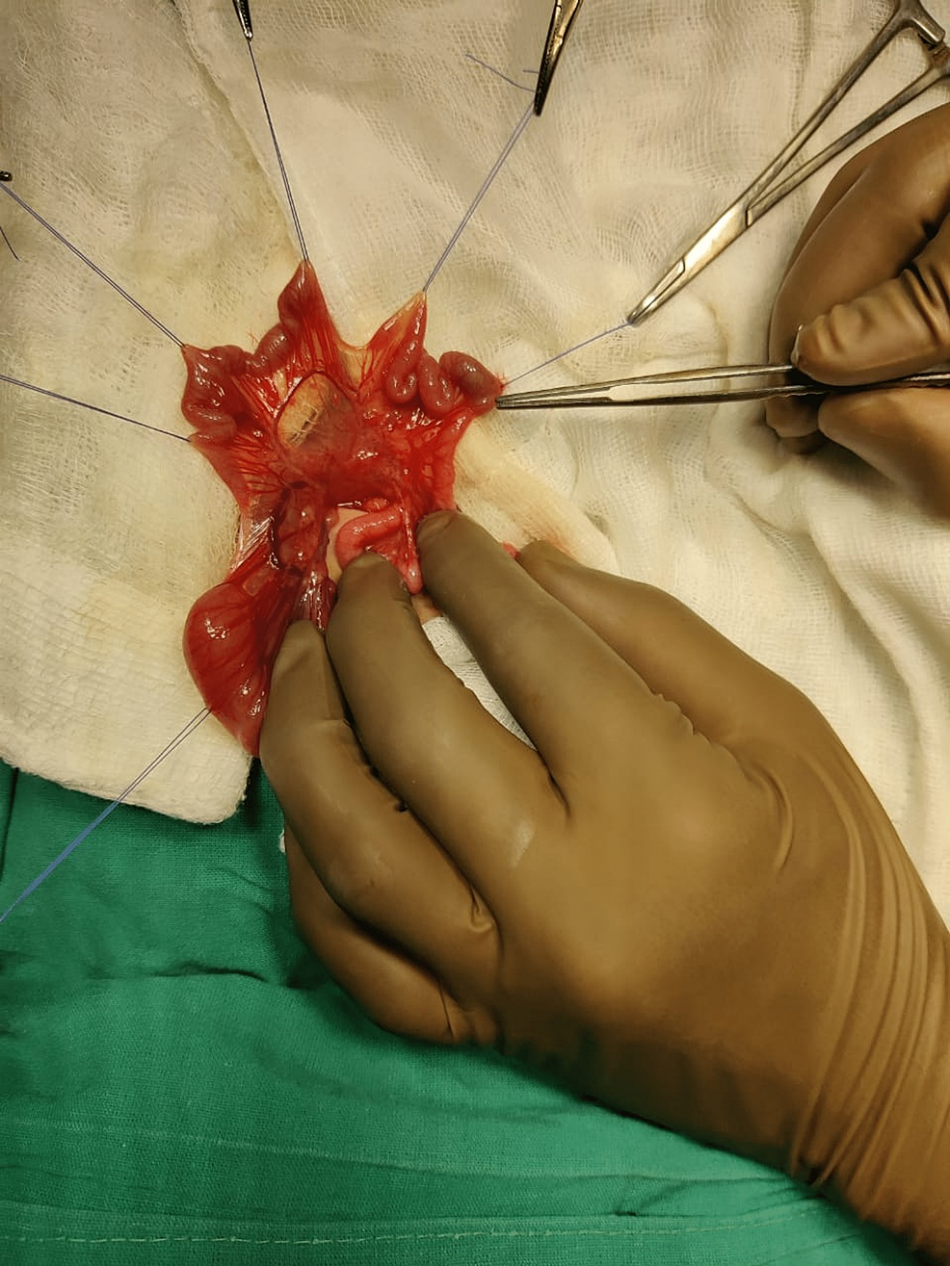
Type IV jejunal atresia.

**Figure 3:**
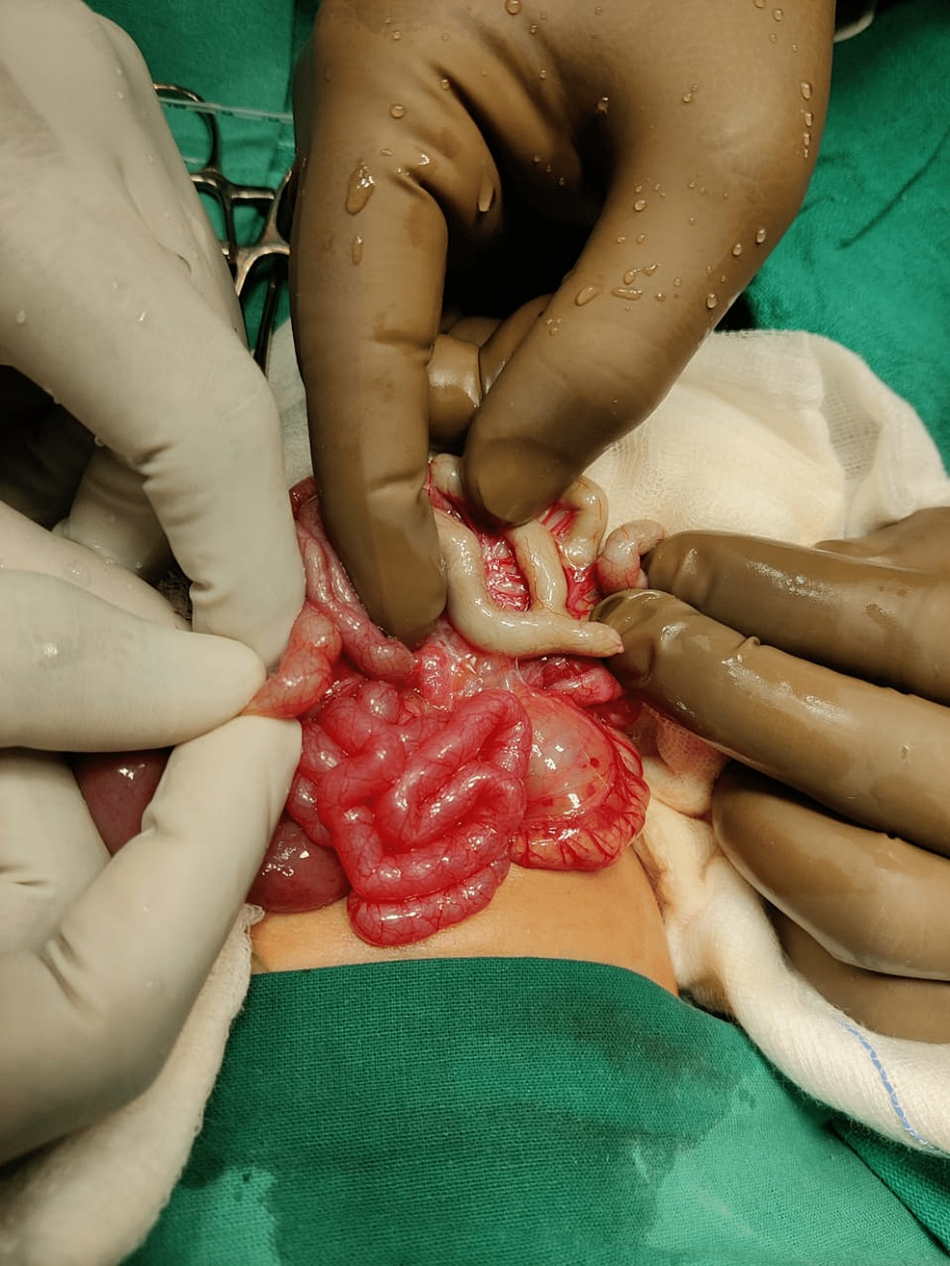
Meckel's diverticulum.

In the current scan, ultrasonography of the abdomen and pelvis revealed dilated large bowel loops with minimal interbowel-free fluid. The baby was then taken for an exploratory laparotomy on an emergency basis. It was discovered intraoperatively that he has type IV jejunal atresia (Figure 2) involving 15 cm from the duodenojejunal flexure and broad-based Meckel’s diverticulum (Figure 3), and he underwent resection of jejunal atretic segments with jejunal end-to-end anastomosis.

Intraoperatively, a 2 cm × 1 cm mass was seen over the early part of the jejunum, which was sent for histological evaluation and found to be histopathologically evocative of the heterotopic pancreas. The baby was started on parenteral nutrition postoperatively, which was well tolerated, and small volume nasogastric feeds were started on postoperative day 7, with no clinically significant complaints. On a postoperative day 12, the feed volume was increased. On postoperative day 15, the baby had one episode of bilious vomiting and a deranged liver function test, after which he started on intermittent infusion feeds.

## Discussion

An ischemic shock to the gut during growth in the delayed intrauterine phase is the most likely cause of bowel atresia. The principal reason for most small bowel and small intestinal atresia is late intrauterine mesenteric vascular impairment. According to reports, volvulus and jejunal atresia can exist side by side, without causing mesenteric vascular irregularities. Bowel atresia can be caused by a variety of factors [10].

In this case, the patient had a heterotopic pancreas, Meckel's diverticulum, atretic jejunum sections, and type IV jejunal atresia. Health conditions for jejunal atresia involve prematurity (50%), polyhydramnios (25%), Crohn's disease (20%), pyloric stenosis, midgut volvulus, and brief gut syndrome [11]. Cigarette smoking, drug habit, and maternal vasoconstrictor drugs may all contribute to the onset of atresia. Type IV jejunal atresia is an inherited autosomal dominant disorder [11].

Other congenital abnormalities affiliated with the annular pancreas in children are well known, with Down's syndrome being the most common birth chromosomal anomaly, with a 10% to 30% occurrence [12]. In addition, cardiac deficiencies, bowel atresia, and small bowel anomalies have been linked to the annular pancreas. Regarding the infant in this case study, while Meckel’s diverticulum is known to be one of the most common congenital malformations, little is known about the potential relationship between the annular pancreas and Meckel's diverticulum, as well as infants who have both annular pancreas and Meckel's diverticulum [12].

The most common symptoms are persistent abdominal cramping, bilious vomiting, and the inability to eliminate meconium. Nevertheless, in addition to the standard underlying diseases of jejunal atresia, characterized by a triple bubble emergence for a proximal blockage, there could be several dilated small intestinal loops anterior to the atresia [13]. The main treatment goals for intestinal atresia are to resect the atretic segment and restore intestinal continuity as much fully operational bowel as possible. The type of operative treatment to be performed is determined by the intraoperative findings [14]. According to Peng et al., end-to-side repair has proven to be both safe and efficient. It also has the advantage of allowing for postoperative GI tract irrigation [15].

## Conclusions

Jejunal webs are far less prevalent than jejunal atresia. These clinical signs can be comparable to those of malrotation and midgut volvulus. The mainstay of planning is early detection and intervention. The surgical strategy is determined by the findings. When the anterior part of the bowel is distended, the first anastomosis is made toward the distal part. To avoid common nutritive complications, primary anastomosis either with or without an enteroplasty is preferred.
